# New Appliances and Things Medical

**Published:** 1898-03-19

**Authors:** 


### NEW APPLIANCES AND THINGS MEDICAL.

[We shall be glad to receive, at our Office, 28 & 29, Southampton Street, Strand, London, W.C., from the manufacturers, specimens of all new preparations and appliances which may be brought out from time to time.]

### FLUID VEJOS.

(Vejos, Limited, 143, West Ham Lane, E.)

Fluid Vejos is a preparation in the form of a fluid extract of meat, which it very much resembles in flavour and appearance. It is, however, stated to be a pure and nutritious vegetable extract, more nutritious and economical than meat extract and readily assimilable on account of its solubility. Analysis showed it to contain 38·2 per cent. solid matter, of which 26 3 per cent. was soluble and 11·9 per cent. insoluble in water, and there was only a very small amount of unaltered starchy matter. It contained 7·3 per cent. mineral constituents. Fluid Vejos is, no doubt, of nutritive value, and a teaspoonful mixed with boiling water as directed made a very palatable cup of Vejos.

### PERFECTED COD LIVER OIL.

(Allen and Hanburys, Limited, Plough Court, Lombard Street, E.C.)

We have on previous occasions made mention in these columns of that excellent preparation of cod-liver oil which is known as "the perfected" brand of Messrs. Allen and Hanburys. Since our last notice the firm have made further improvements in its manufacture, and now more than ever it deserves its descriptive title. It is more nearly tasteless than before, and leaves the mouth comparatively free from traces of its presence a few moments after it has been swallowed. It appears completely free from nauseous oxidation products, is easily assimilated, and hence less likely to repeat than many other preparations which do not fulfil the above conditions.

### VERONI.

(Williams and Co., Limited, 50 and 52, Commercial Street, London, E.)

Veroni, which is a most excellent wheat preparation, containing a large percentage of flesh-formers in the form of albuminoids, is a most carefully prepared preparation. It constitutes a capital ingredient for the making of various cakes, puddings, custards, &c. It has, further, the advantage of being a pure product of careful and cleanly manufacture, and costs no more than 4d. a pound. In the preparing of novel and pleasant invalid delicacies it will be found invaluable to the cook. The manufacturers have made a further and valuable development of its sphere of usefulness in rendering it a basis for the manufacture of vermicelli and macaroni. These indispensable articles of high-class cookery usually deserve the reproach imputed to them—that there has been some want of care in attention to details of cleanliness in their preparation. The macaroni and vermicelli prepared by Messrs. Williams from their well-known Veroni can be relied upon as a pure and cleanly article of manufacture, and for this reason will be of special value for sick-room cookery.

### DIASTOL.

(The Standard Malt Extract Company, Limited, 21, Water Street, Liverpool; and 23, Billiter Street, London, E.C.)

Diastol has now become a familiar adjunct to the natural processes of digestion among invalids. Unlike ordinary malt extract, it does not contain a large percentage of converted carbo-hydrates, and hence must not be regarded as a food. Its diastatic power or power of digesting amylaceous or starchy food are exceedingly great, and hence for those persons who cannot rely on their own natural digestive powers to perform these functions, diastol will be found of the greatest use. It is a carefully-made product, and can be relied upon to perform the function of an artificial digestant in the most feeble or emaciated.

### HORLICK'S MALTED MILK.

(Horlick's Food Co., 34, Farringdon Road, London, E.C.)

Horlick's malted milk has been before this the subject of a short notice in our columns. Since then we have made a more extensive trial of it as a food for infants and for invalids. There can be no doubt that it is in every way a most desirable addition to the nursery and invalid dietary when other foods defy digestion and absorption. It is always readily taken, and in no case where any form of fluid can be tolerated by the stomach have we found it rejected. The only objection to its use that we discover is that its very ease of administration may perhaps lead to a habit of substituting it for the more natural and simple foods.

